# Supplementary material for: Defect-driven nanostructuring of low-nuclearity Pt-Mo ensembles for continuous gas-phase formic acid dehydrogenation
Source: Nat Commun. 2023 Nov 18;14:7518. doi: 10.1038/s41467-023-42759-5 (PMC10657381; doi:10.1038/s41467-023-42759-5)
Supplement: Supplementary file 1 — Supplementary Information [file 41467_2023_42759_MOESM1_ESM.pdf]

Supplementary Information

Defect-driven nanostructuring of low-nuclearity Pt-Mo ensembles for continuous gas-phase formic acid dehydrogenation

Luyao Guo,[1,2#] Kaixuan Zhuge,[3#] Siyang Yan,[4#] Shiyi, Wang,[1] Jia Zhao,[3*] Saisai Wang,[3] Panzhe Qiao,[5] Jiaxu Liu,[4*] Xiaoling Mou,[1,6] Hejun Zhu,[2*] Ziang Zhao,[2] Li Yan,[2] Ronghe Lin,[1,6*] and Yunjie Ding[1,2,7*]

[1]Hangzhou Institute of Advanced Studies, Zhejiang Normal University, 1108 Gengwen Road, Hangzhou 311231, China.

[2]Dalian National Laboratory for Clean Energy, Dalian Institute of Chemical Physics, Chinese Academy of Sciences, 457 Zhongshan Road, Dalian 116023, China.

[3]Institute of Industrial Catalysis, Zhejiang University of Technology, Hangzhou, 310014, China

[4]Department of Catalytic Chemistry and Engineering & State Key Laboratory of Fine Chemicals, Dalian University of Technology, Dalian 116012, China.

[5]Shanghai Synchrotron Radiation Facility, Zhangjiang Lab, Shanghai Advanced Research Institute, Chinese Academy of Sciences, Shanghai 201204, PR China

[6]Key Laboratory of the Ministry of Education for Advanced Catalysis Materials, Zhejiang Normal University, 688 Yingbin Road, Jinhua 321004, China.

[7]The State Key Laboratory of Catalysis, Dalian Institute of Chemical Physics, Chinese Academy of Sciences, 457 Zhongshan Road, Dalian 116023.

Emails: jiazhao@zjut.edu.cn, liujiaxu@dlut.edu.cn; zhuhj@dicp.ac.cn, catalysis.lin@zjnu.edu.cn, dyj@dicp.ac.cn

[#]These authors contributed equally.

Supplementary Table 1. Comparison on the performance between Pt-Mo/$NC_{0.07}$ and the literature reported solid catalysts in the gas- and liquid-phase dehydrogenation of formic acid.

Catalysts	Precious metal wt.%	Feed	50% conversion T / K	50% conversion S_{H2} / %[a]	TOF at 373 K / s^{-1}	rate[b] s^{-1}	E_a / kJ mol^{-1}	Stability r_d / h^{-1} [c]	Stability T / K	Stability Duration / h	refs.
**Gas-phase dehydrogenation**											
Pd-K/C	1.0	2.0%FA-He	345	99	-	-	97	-	-	-	1
Pd/N-CNFs	1.0	1.9% FA-He	427	~97.6	0.21(398K)	0.063	-	-	-	-	2
Pd/N-PCN	1.0	1.9%FA-He	422	~95.6	0.27(398K)	0.049	-	–0.33	448	30	2
Pd/Mel/C	1.0	2.5%FA-Ar	528	>98	-	0.079(393K)	32	–1.5	573	6	3
Pt/N-CNFs	1.0	2.0%FA-He	445	99.5	0.088	-	43-53	-	-	-	4
Pt/N-CNFs	0.3	1.8%FA-He	469	99.6	0.25(398K)	0.038(398K)	-	–0.14	448	42	5
Ir/AC	2.0	5.0%FA-Ar	383	100	0.096	0.025	-	-	-	-	6
Pt/AC	2.0	5.0%FA-Ar	423	98	0.064	0.015	-	-	-	-	6
Pt/N-graphene	1.0	5.0% FA-He	443	98	0.42(398K)	0.025	-	0	423	5	7
Au/Al_2O_3	2.5	1.9%FA-He	456	99.6	-	0.0072	57	–0.43	423	16	8
Mo_2C/AC	0	5.5%FA-N_2	423	95	0.066	-	-	0	453-493	12	9
Mo_2C-Co/AC	0	-	445	99.5	0.088	-	-	-	-	-	10
Pt-Mo/$NC_{0.07}$	0.48	4.15%FA-H_2O-N_2-He	360	>99.9	0.31	0.31	54	0	373	50	*
					0.62(388K)	0.62(388K)					*
**Liquid-phase dehydrogenation**											
Catalysts	Precious metal wt.%	Feed	T / K	CO / ppm	TOF / s^{-1}	rate[b] s^{-1}	E_a / kJ mol^{-1}				refs.

Pd/S-1-in-K	0.64	FA-SF-H_2O	323	<10	0.84	0.91	39	11
in situ-Pd/MSC	4.6	FA-SF-H_2O	333	n.d.	2.53	4.44	31	12
Pd_1Au_1/30-LA	3.4Au, 1.8Pd	FA-SF-H_2O	333	n.d.	2.32	-	39	13
Pd@CN900K	9.3	FA-SF-H_2O	333	<10	4.00	-	47	14
Pd/BNF-C	9.1	FA-SF-H_2O	313	n.d.	1.06	-	36	15
(Co-N)6.8@NC	6.8	FA-propylene carbonate	393	n.d.	-	0.012	47	16
PdNi-WO_x/KIT-6-NH_2	9.98Pd	FA-H_2O	323	n.d.	1.22	-	35	17
HCOONa-Pd@ANI/C	3.74	FA-SF-H_2O	303	n.d.	-	0.58	-	18
Pd-WO_x/(P)NPCC	14.3Pd	FA-SF-H_2O	323	n.d.	1.70	-	36	19
γ-Mo_2N/0.2NK-C	26Mo	FA-H_2O	360	n.d.	-	$1.4x10^{-5}$	-	20

Note: $^a H_2$ selectivity. ^{b}rate in mol of HCOOH converted per mol of precious metals per second. ^{c}Deactivation rate defined by the change in HCOOH conversion in percentage per hour during stability tests. *This work. FA: formic acid, SF: sodium formate. n.d.: not detected.

Supplementary Table 2. Characterization data of the NC hosts and the corresponding metal-supported catalysts.

Sample	V_{total}^{a} / (cm^3 g^{-1})	S_{BET}^{b} / (m^2 g^{-1})	C^{c} / at.%	N^{c} / at.%	Pt^{d} / wt.%	Mo^{d} / wt.%	$N:C^{e}$ / mol mol^{-1}
$NC_{0.13}$	0.32	491	62.17	9.42	-	-	$0.13(0.15)^{c}$
$NC_{0.07}$	0.26	464	74.86	5.93	-	-	$0.07(0.08)^{c}$
$NC_{0.02}$	0.38	571	88.42	2.06	-	-	$0.02(0.02)^{c}$
Pt/$NC_{0.07}$	0.90	182	-	-	0.54	-	-
Mo/$NC_{0.07}$	0.40	347	-	-	-	5.56	-
Pt-Mo/$NC_{0.07}$	0.72	227	-	-	0.48	5.04	-
Pt-Mo/$NC_{0.13}$	0.73	190	-	-	0.46	4.87	-
Pt-Mo/$NC_{0.02}$	0.34	288	-	-	0.43	4.84	-

[a]Determined from the amount of N_2 adsorbed at $p/p_0 = 0.97$. [b]BET method. [c]XPS. [d]ICP-OES. [e]C,H,N elemental analysis.

Supplementary Table 3. EXAFS fitting parameters at the Pt L_3-edge and Mo *K*-edge of the key catalysts and the reference Pt and Mo foils.

Sample	Path	CN^{a}	R / Å ^{b}	σ^2 / Å$^{2\ c}$	ΔE_0 / eV ^{d}	R factor^{e}
Pt foil	Pt-Pt	12	2.765	0.004	-0.003	0.0078
Mo foil	Mo-Mo	8	2.72	0.003	0.002	0.1
Pt/NC$_{0.07}$	Pt-N	5.05	2.07	0.009	0.11	0.014
Mo/NC0.07	Mo-O	0.95	1.874	0.007	-0.154	0.05
	Mo-N	2	2.176	0.005	-0.301	
Pt-Mo/NC$_{0.07}$	Pt-N	3.1	2.03	0.002	-0.072	
	Pt-Pt	1.1	2.74	0.001	0.029	
	Pt-Mo	1.8	2.45	0.006	-0.023	
	Mo-O	0.8	1.874	0.002	-0.166	
	Mo-N	1.1	2.176	0.004	-0.231	0.01
	Mo-Pt	1	2.422	0.004	-0.176	

Note: ^{a}Coordination numbers, ^{b}bond distance, ^{c}Debye-Waller factors, ^{d}the inner potential correction, ^{e}goodness of fit. The experimental EXAFS fit of metal foils by fixing *CN* as the known crystallographic values.

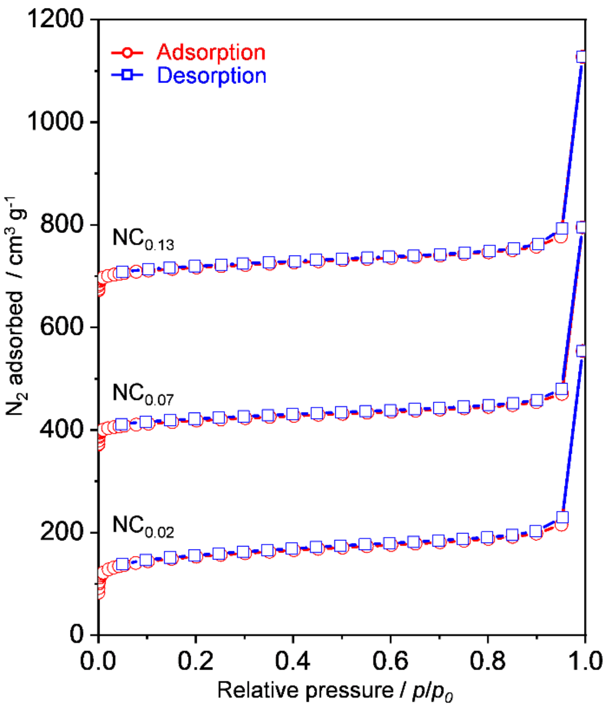

Supplementary Fig. 1. N_2 sorption isotherms of the N-doped carbon with different N:C ratios. The isotherms were stacked by upshift by 300 $cm^3\ g^{-1}$ for each catalyst from the bottom to the top.

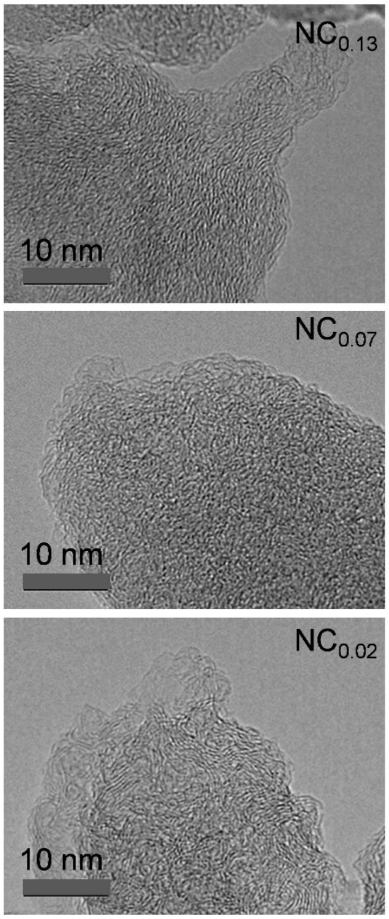

Supplementary Fig. 2. TEM images of the N-doped carbon with different N:C ratios.

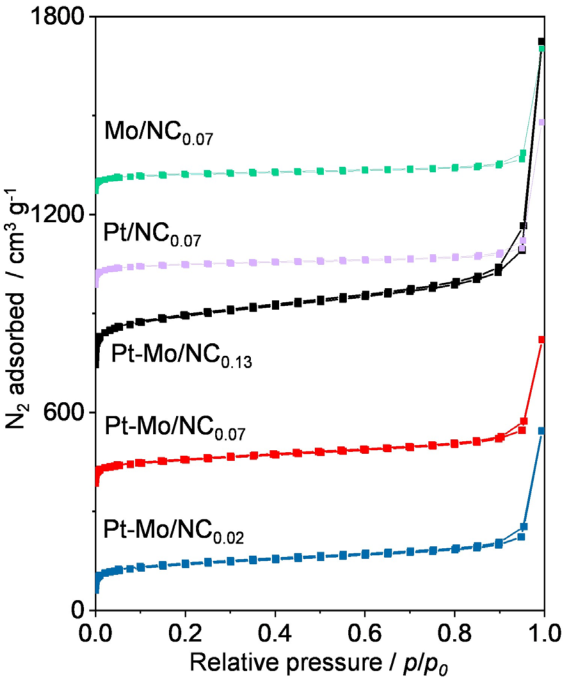

Supplementary Fig. 3. N_2 sorption isotherms of the mono- and bi-metallic catalysts. The isotherms were stacked by upshift by 300 cm^3 g^{-1} for each catalyst from the bottom to the top.

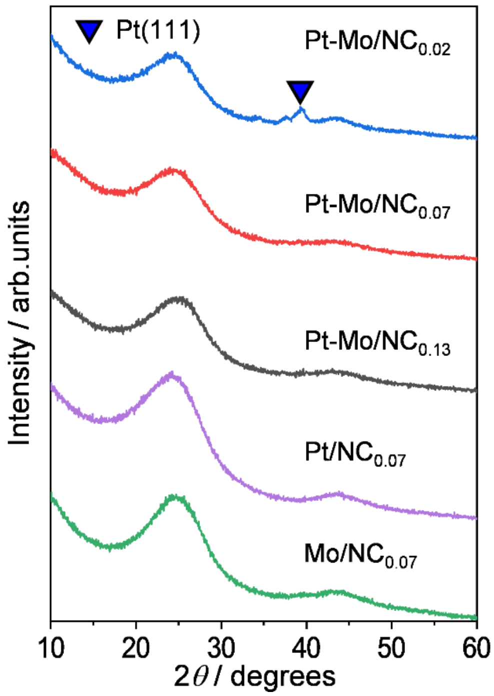

Supplementary Fig. 4. The PXRD patterns of the mono- and bi-metallic catalysts.

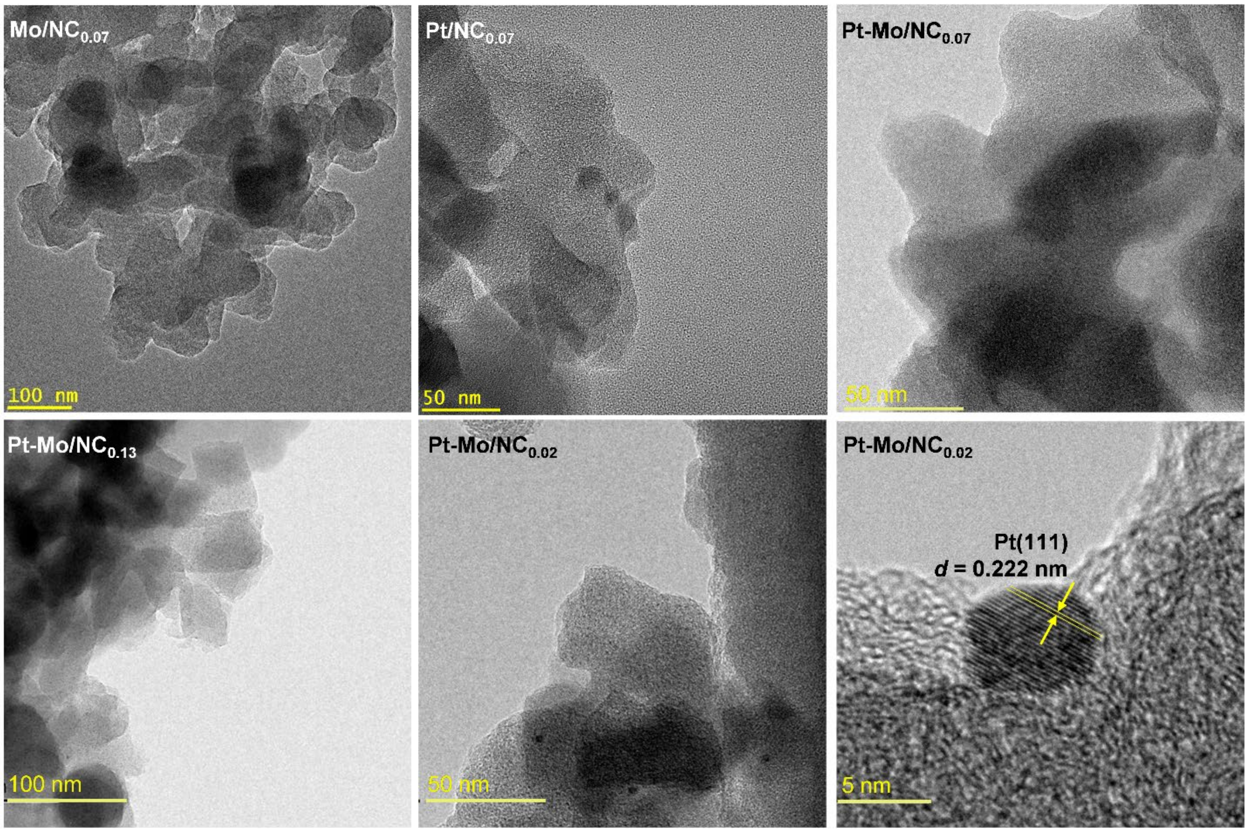

Supplementary Fig. 5. TEM images of the representative mono- and bi-metallic catalysts.

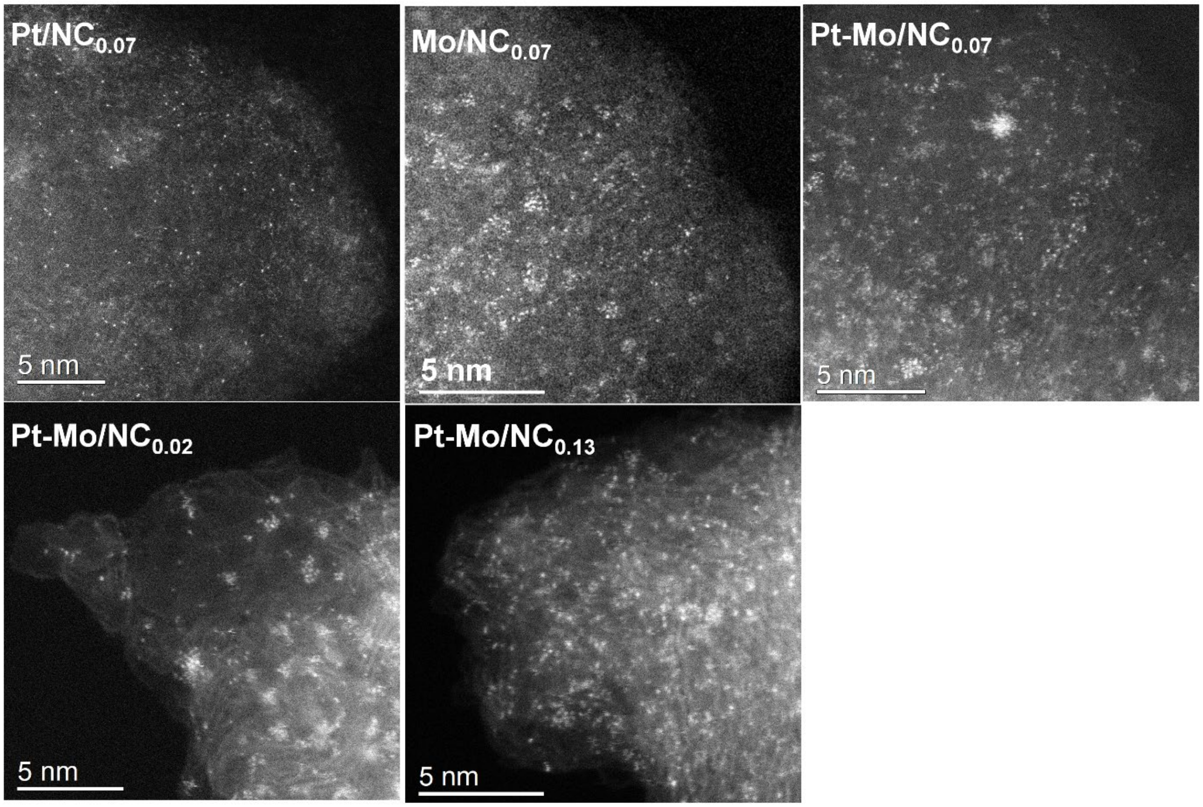

Supplementary Fig. 6. Additional HAADF-STEM images of the representative mono- and bi-metallic catalysts.

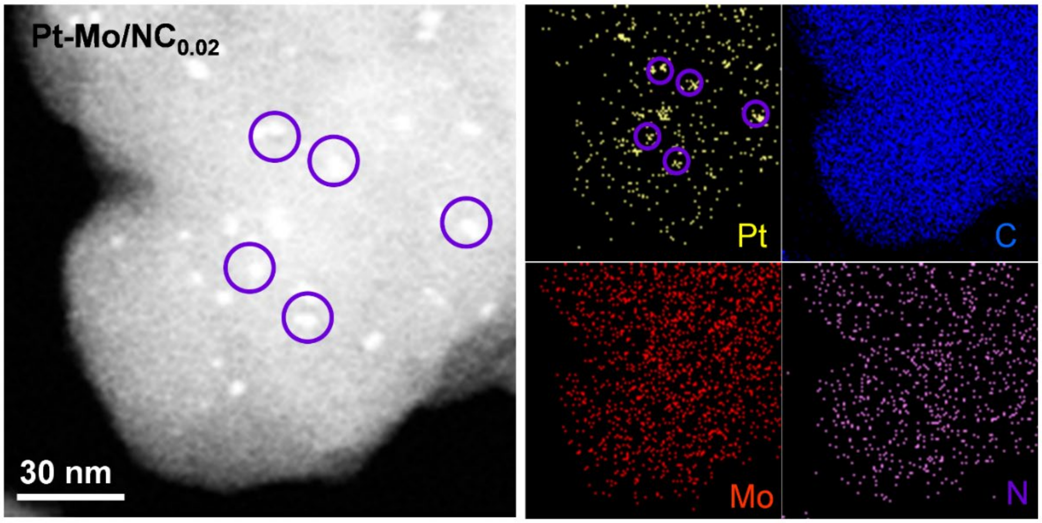

Supplementary Fig. 7. HAADF-STEM images with elemental color mapping of Pt-Mo/NC$_{0.02}$. Small particles highlighted by the circles evidenced the aggregation of Pt species on N-doped carbon with a low N:C ratio.

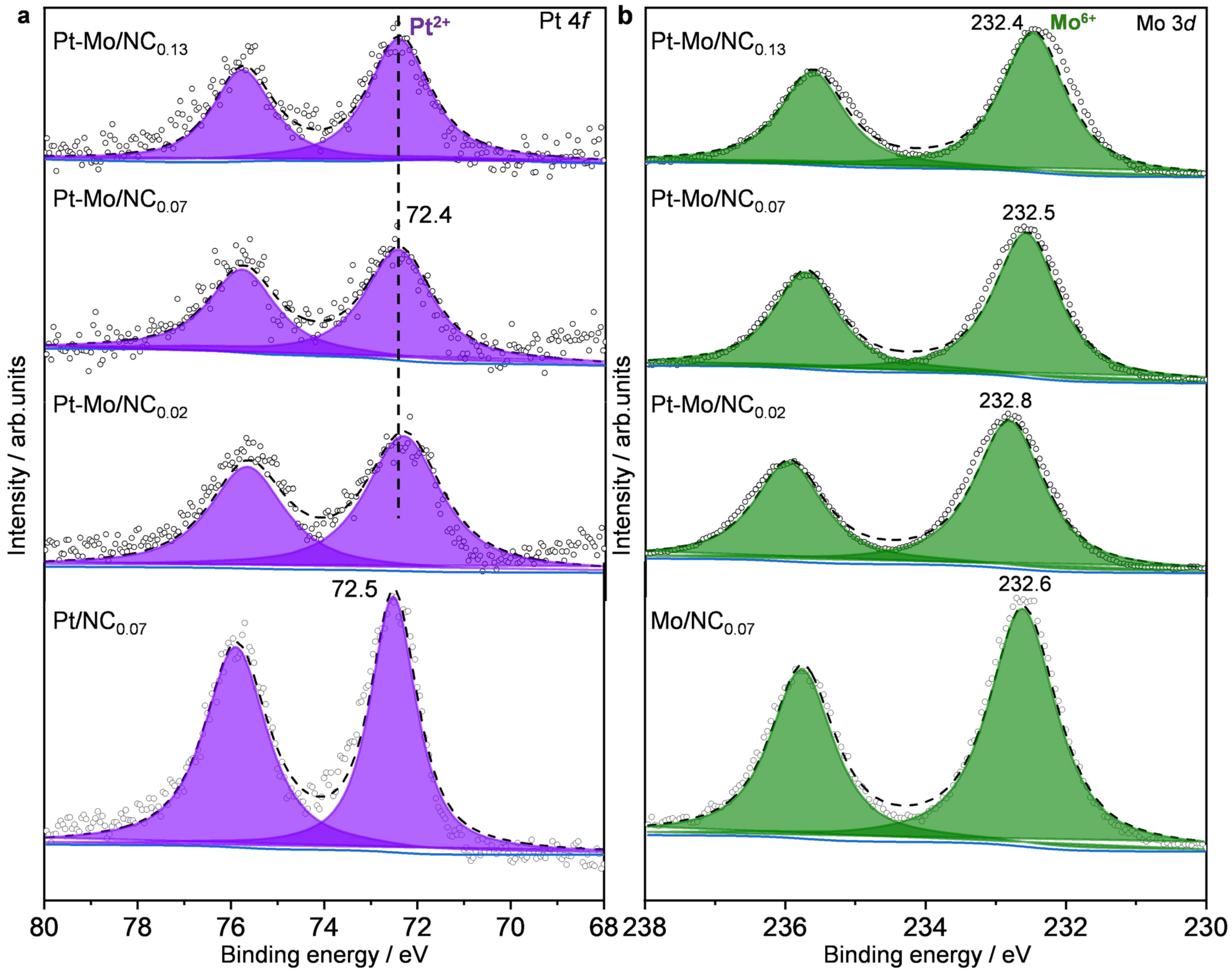

Supplementary Fig. 8. The core-level XPS spectra of the mono- and bi-metallic catalysts. a, Pt 4*f*, **b**, Mo 3*d*. The purple and green shadows show the fitted doublets for Pt^{2+} and Mo^{6+} species, respectively. The cycles represent the raw XPS data.

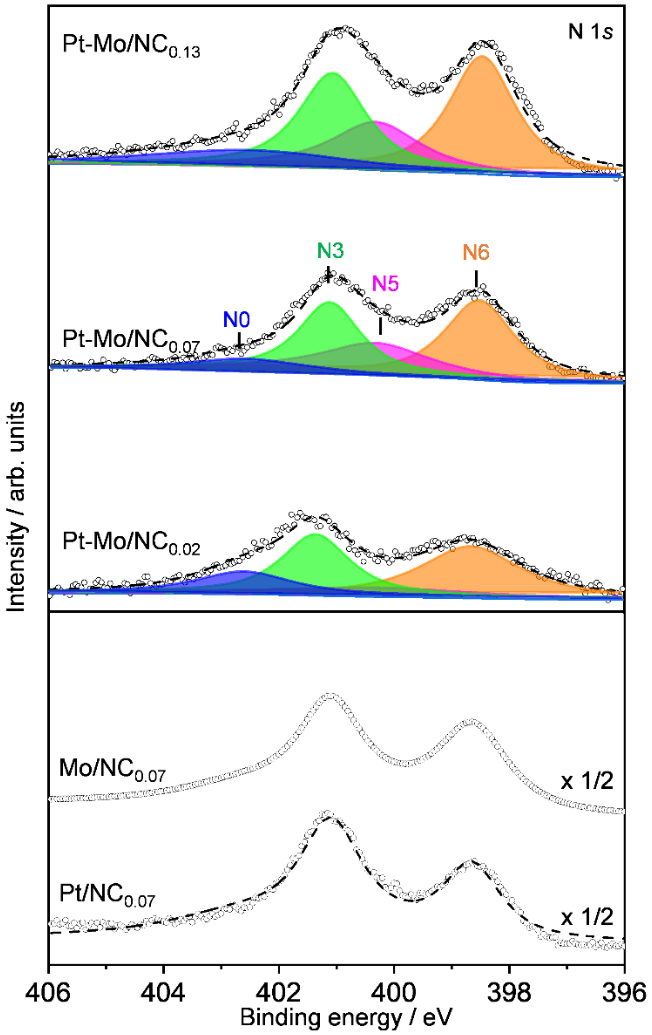

Supplementary Fig. 9. The core-level N 1*s* XPS spectra of the mono- and bi-metallic catalysts. N6, N5, N3, and N0 represented the pyridinic, pyrrolic, quaternary, and oxidized pyridinic N, respectively. The shadows show the fitted peaks of different nitrogen species. The cycles represent the raw XPS data.

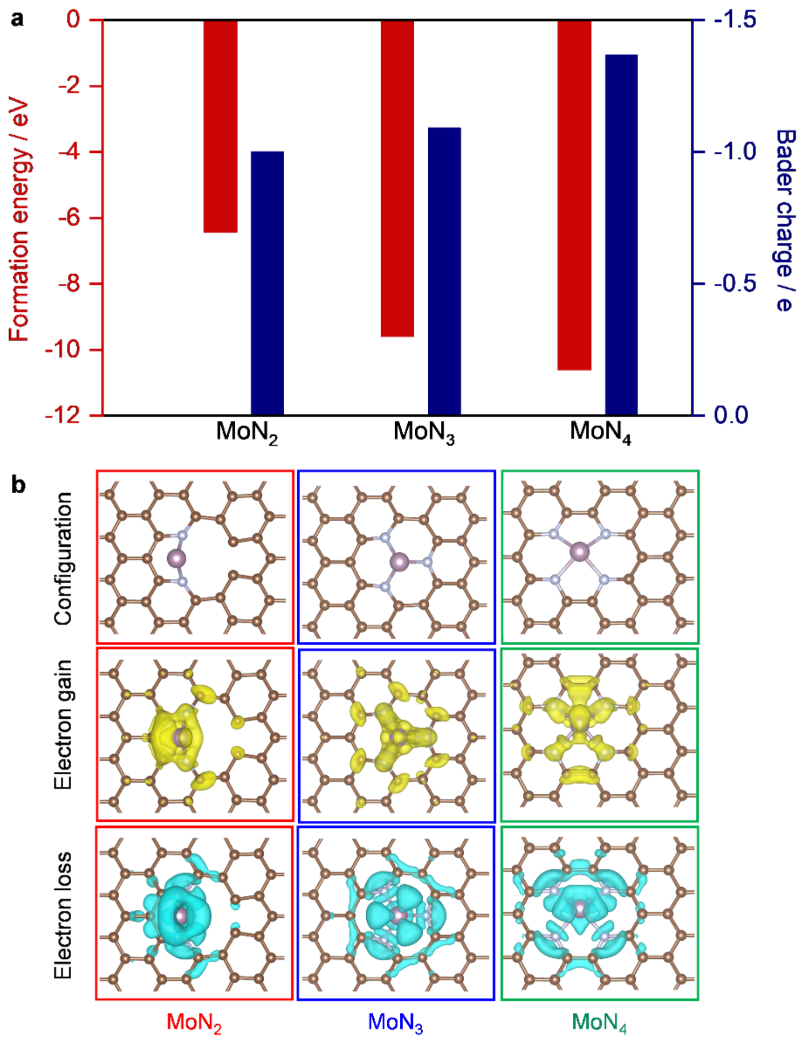

Supplementary Fig. 10. **Formation energy and Bader charge analysis of different MoN_x (x = 2-4) entitles. a**, The formation energy and Bader charge, and, **b**, the configurations and charge density plots of MoN_x.

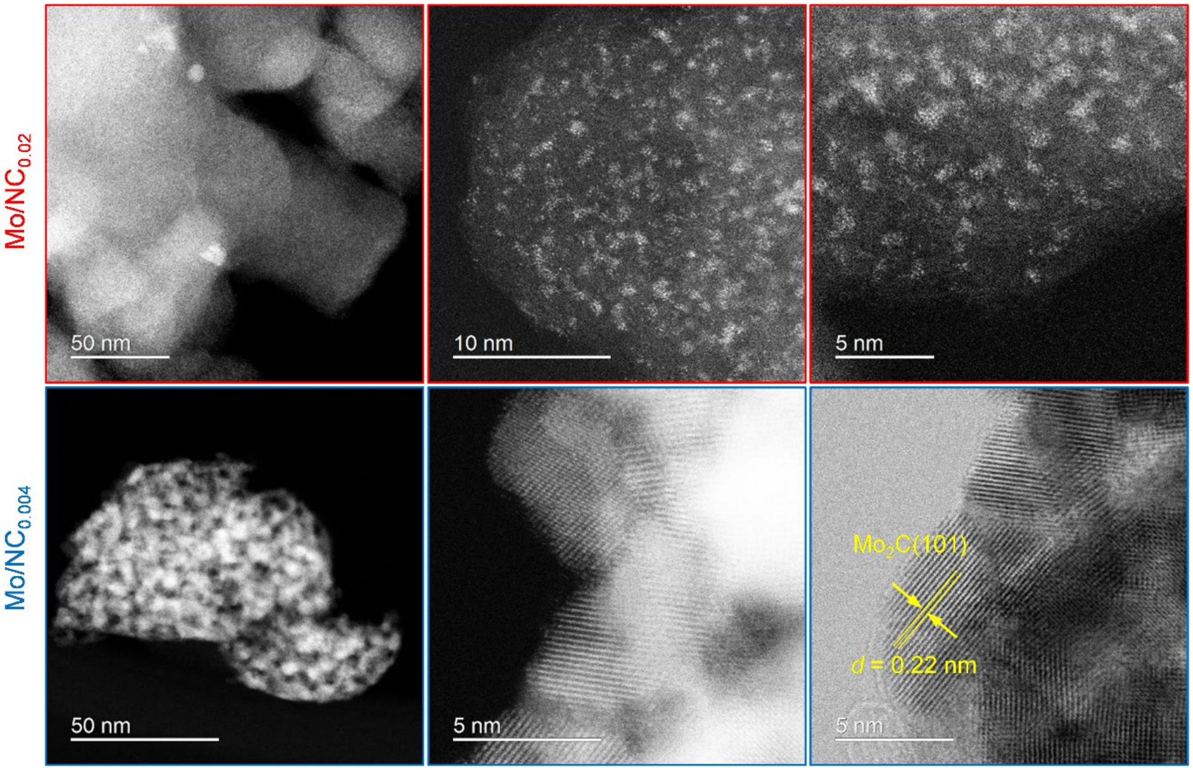

Supplementary Fig. 11. **The HAADF-STEM images of Mo/NC$_{0.02}$ and Mo/NC$_{0.004}$.**

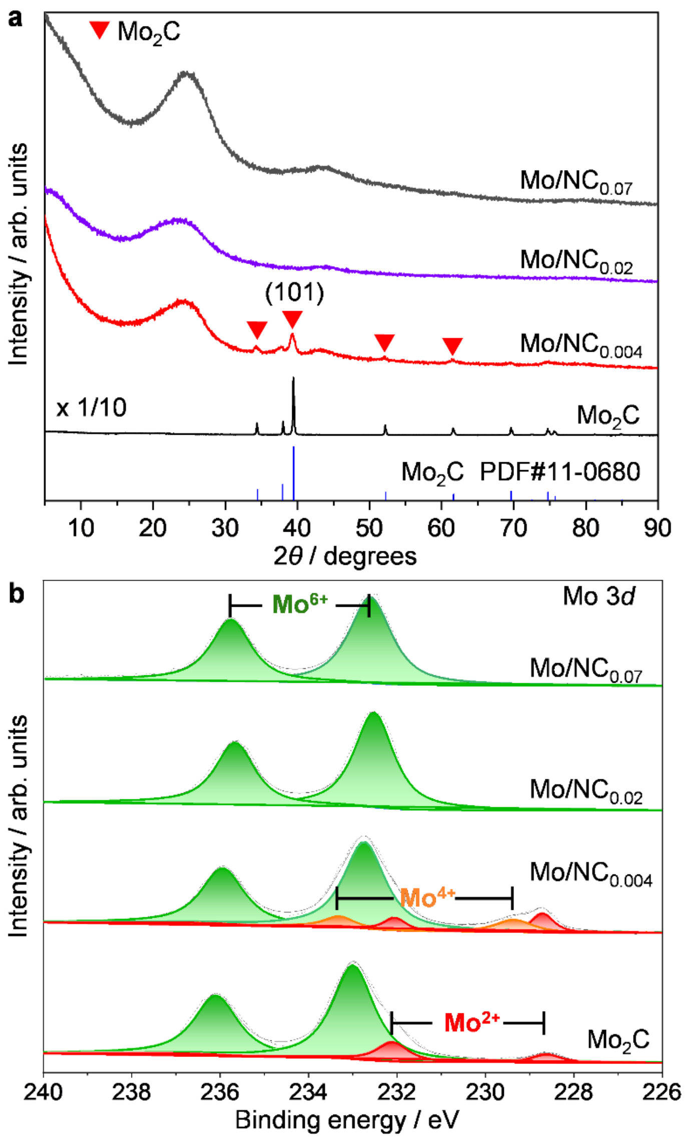

Supplementary Fig. 12. **Characterizations of Mo-based catalysts.** **a**, PXRD and, **b**, Mo 3*d* XPS spectra of Mo/NC$_x$ and commercial Mo_2C. The vertical lines in **a** show the reference standard of Mo_2C. The green, orange, and red shadows show the fitted doublets of Mo^{6+}, Mo^{4+}, and Mo^{2+} species, respectively.

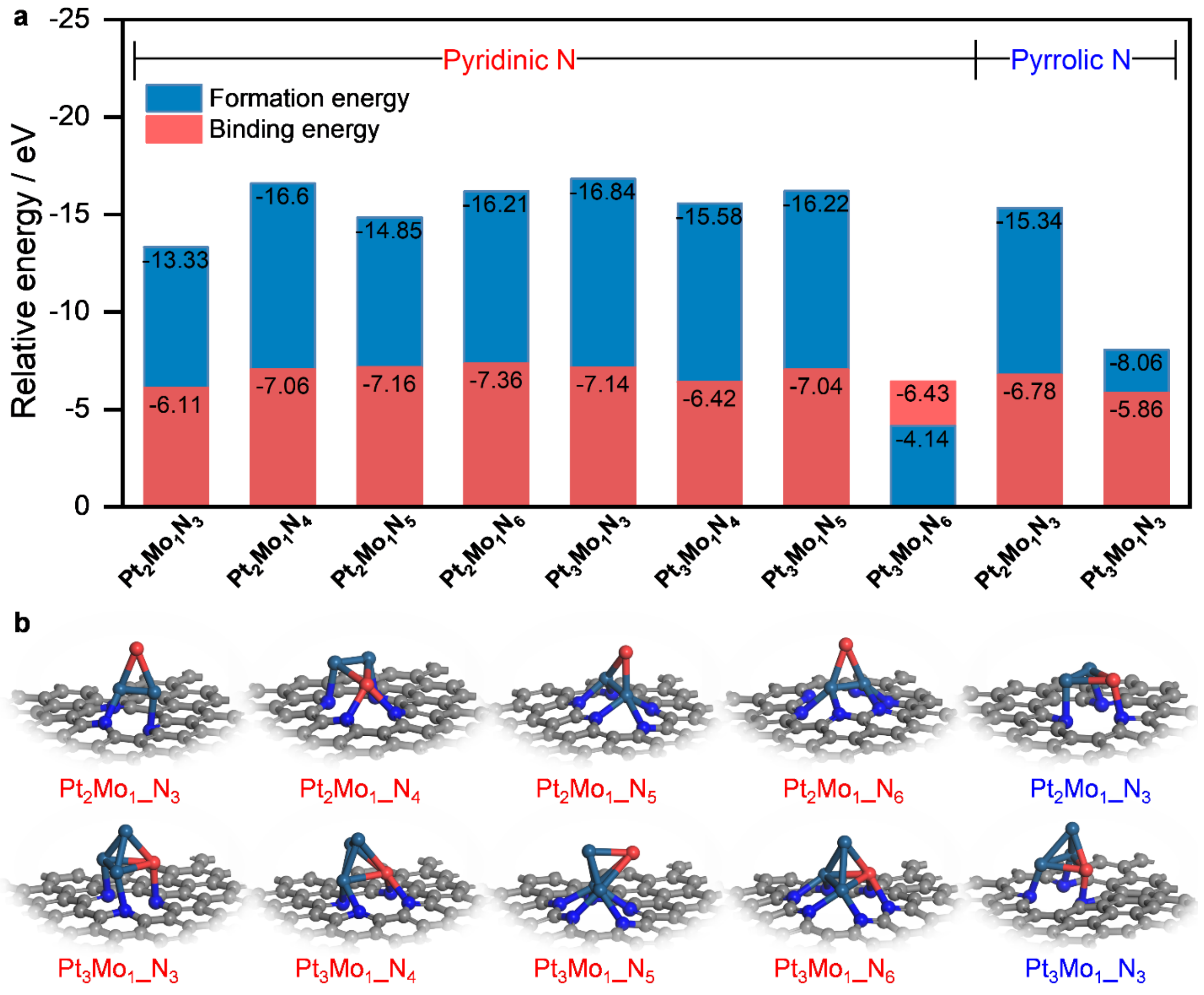

Supplementary Fig. 13. Screening of the possible configurations of the metal ensembles in Pt-Mo/NC0.07. a,The formation energy of the metal clusters and their binding energy on various defects, accompanied with **b**, their configurations. The pyridinic- and pyrrolic-N defect-stabilized clusters were highlighted in red and blue, respectively, in **b**. The balls of grey, blue, light blue, and red represent C, N, Pt, and Mo atoms, respectively.

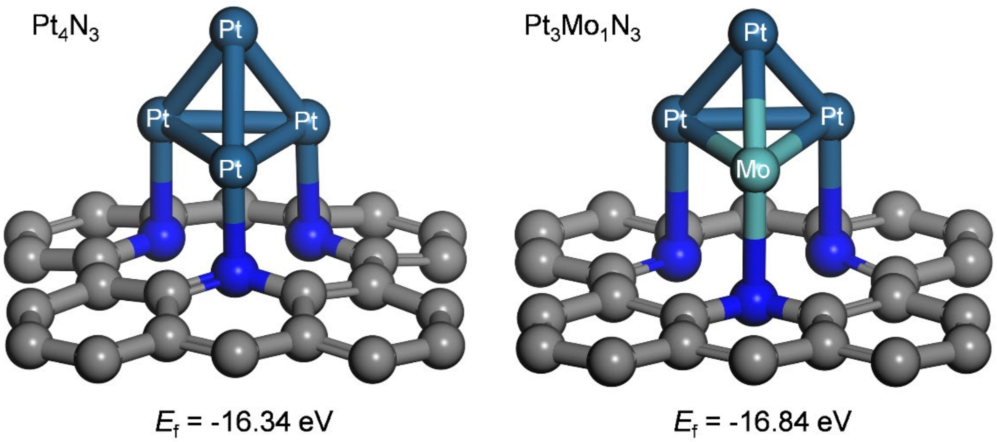

Supplementary Fig. 14. **The configurations of Pt_4N_3 and $Pt_3Mo_1N_3$, accompanied with the formation energy (E_f).**

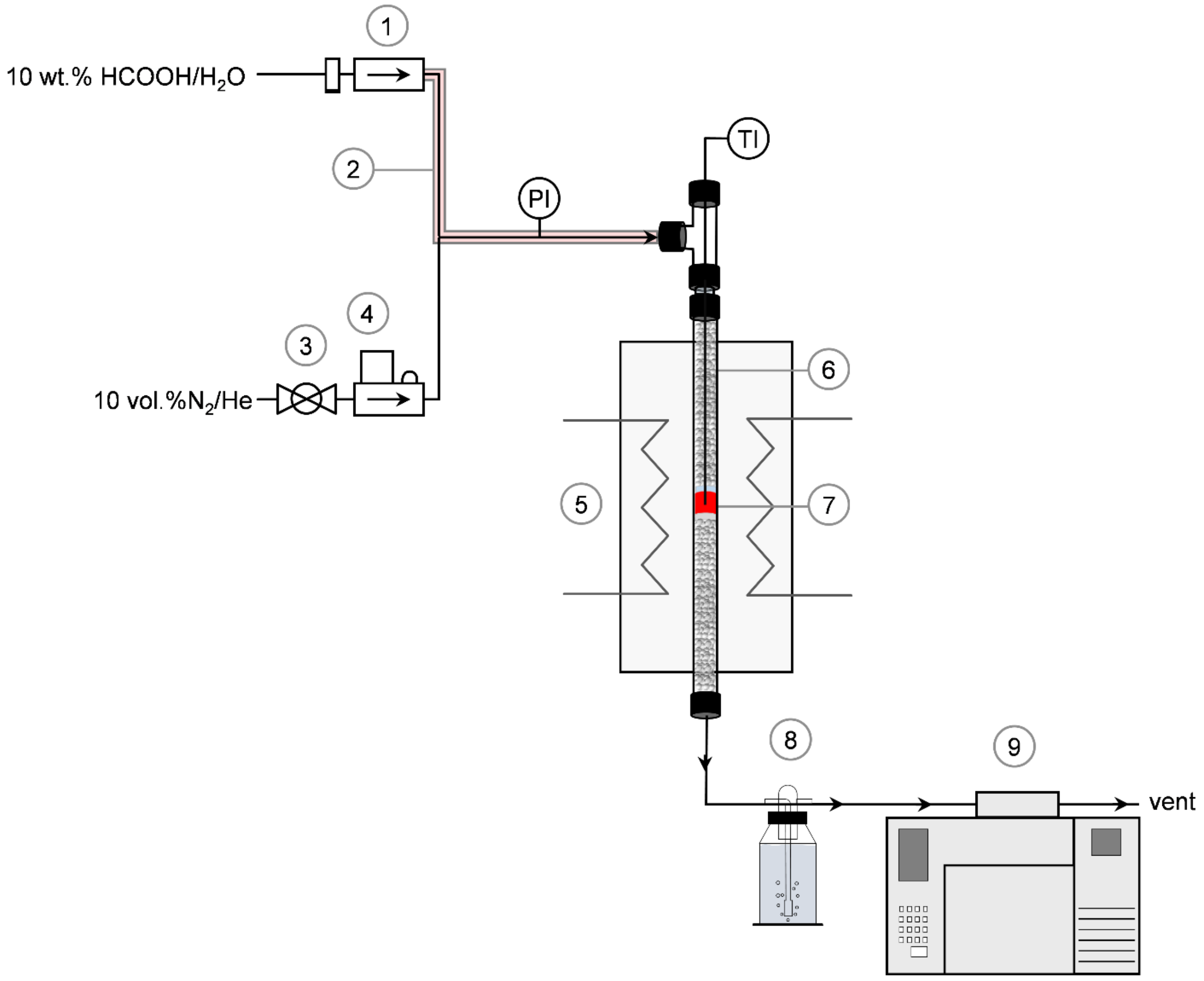

Supplementary Fig. 15. **The flowsheet of the laboratory set-up for the gas-phase dehydrogenation of formic acid.** ① syringe pump, ② heat tracing, ③ two-way on-off valves, ④ mass flow controllers, ⑤ oven, ⑥ quartz reactor, ⑦ catalyst bed, ⑧ cold trap for water removal, ⑨ GC, PI: pressure indicator, and TI: temperature indicator.

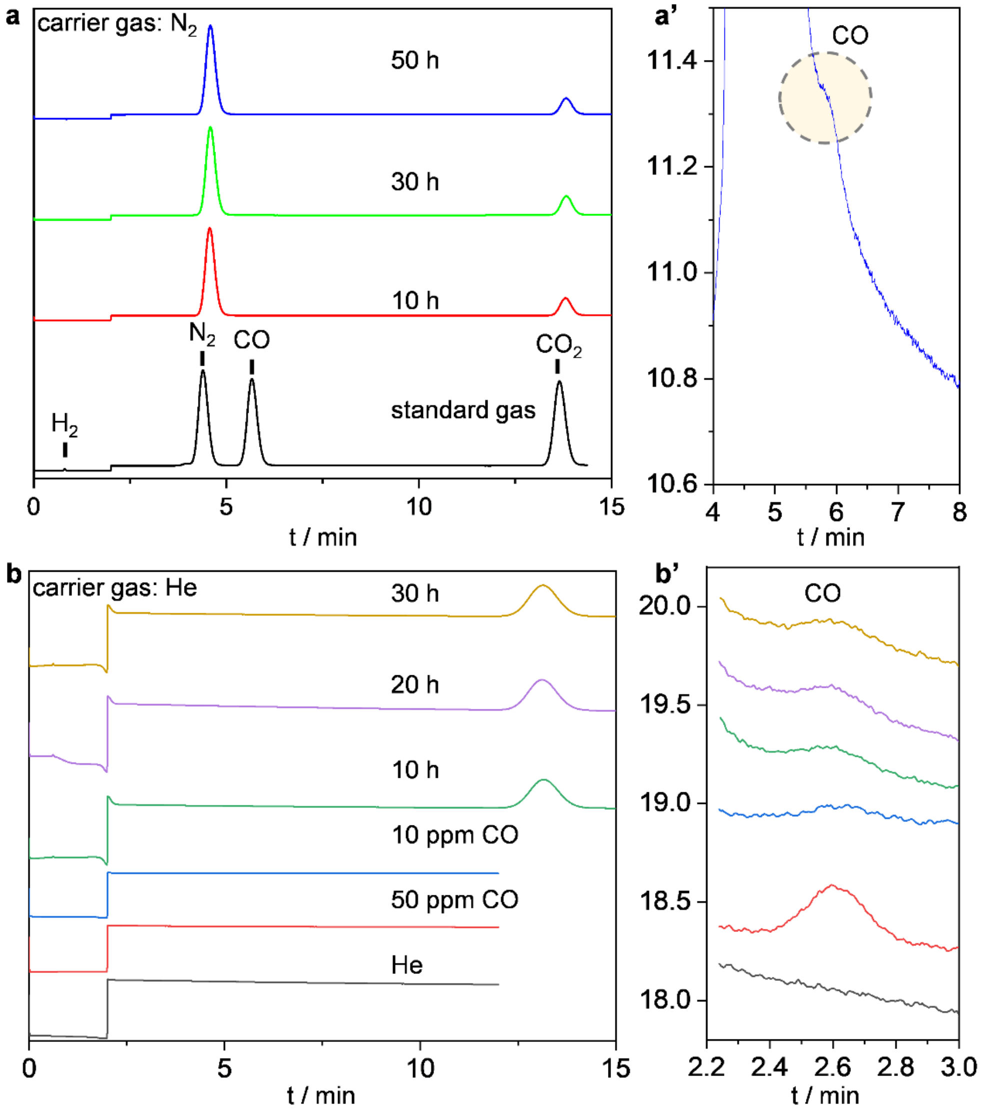

Supplementary Fig. 16. **Analysis of the gaseous reaction products.** The gas chromograph (GC) profiles of the products in HCOOH decomposition on Pt-Mo/$NC_{0.07}$ during stability test, together with the profiles of standard gases (10 and 50 ppm CO/He). Carrier gases used in the GC analysis: **a**, N_2, **b**, He. **a**' and **b**' are the zoomed profiles in **a** and **b**, respectively.

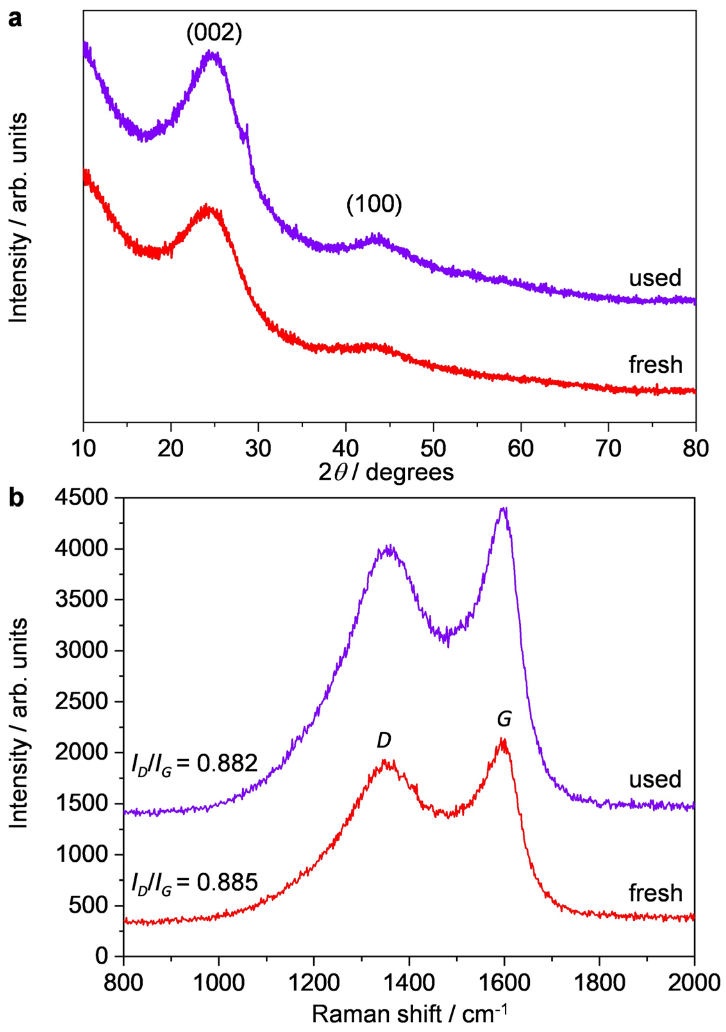

Supplementary Fig. 17. Comparison on the characterization results between the fresh and the used Pt-Mo/$NC_{0.07}$. a, PXRD patterns and, **b**, Raman spectra of Pt-Mo/$NC_{0.07}$ after the 50 h stability test in formic acid decomposition. The ratios of the intensities of the D and G bands (I_D/I_G) were listed in **b**.

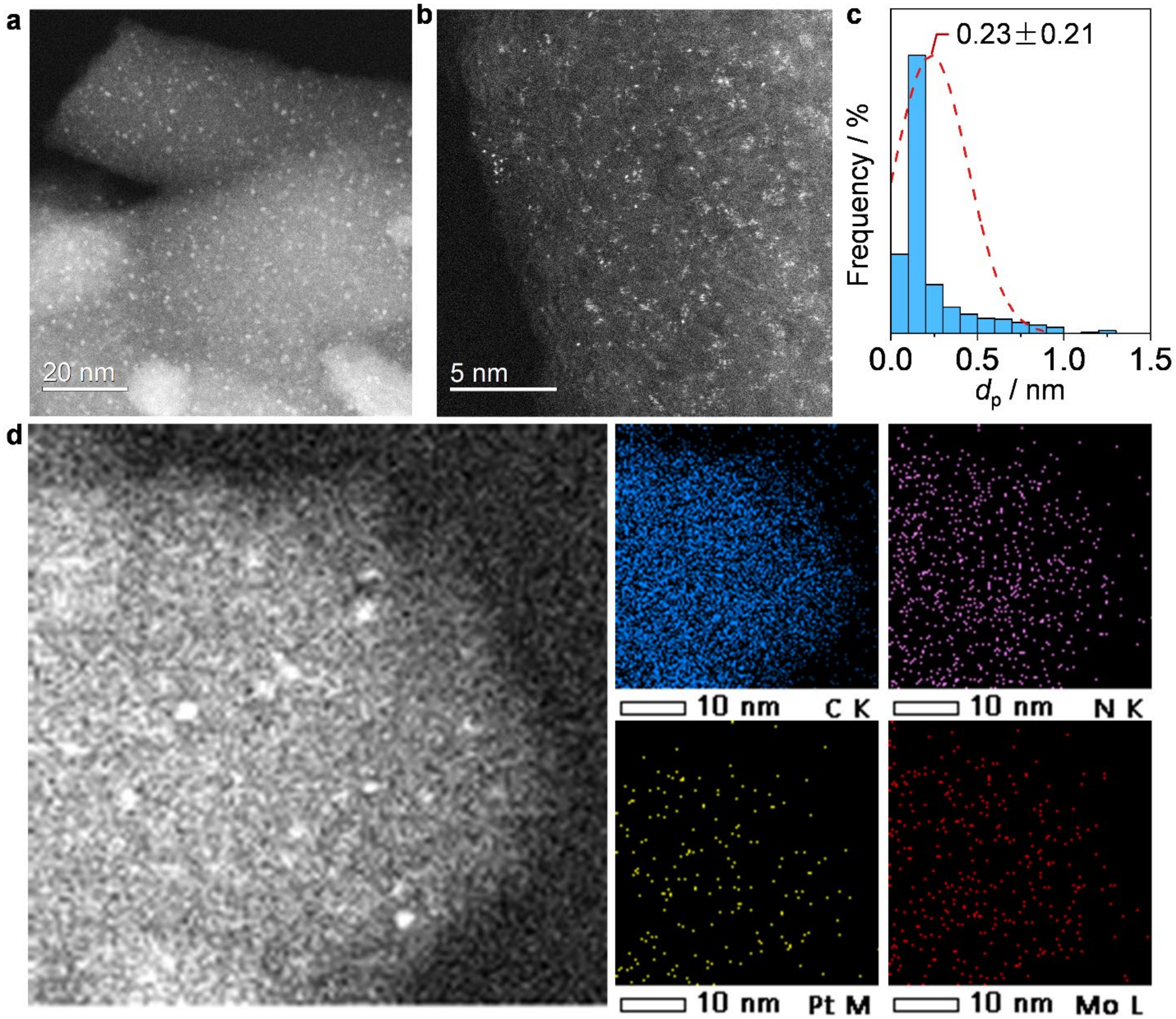

Supplementary Fig. 18. Electron microscopic analysis of Pt-Mo/NC$_{0.07}$ after the stability test. a,**b**, HAADF-STEM images with **c**, particle size distribution, and **d**, elemental color mapping of Pt-Mo/NC$_{0.07}$ after the 50 h stability test in formic acid decomposition.

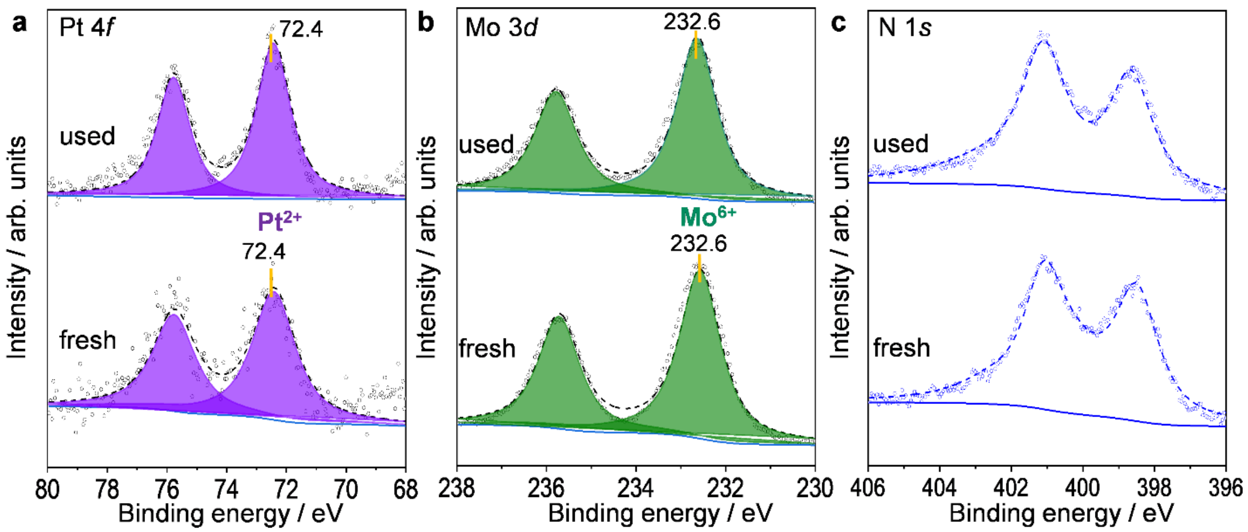

Supplementary Fig. 19. Comparison on the core-level XPS spectra of Pt-Mo/NC$_{0.07}$ before and after the stability test. a, Pt 4*f*, **b**, Mo 3*d*, **c**, N 1*s*. The purple and green shadows show the fitted doublets for Pt^{2+} and Mo^{6+} species, respectively. The cycles represent the raw XPS data.

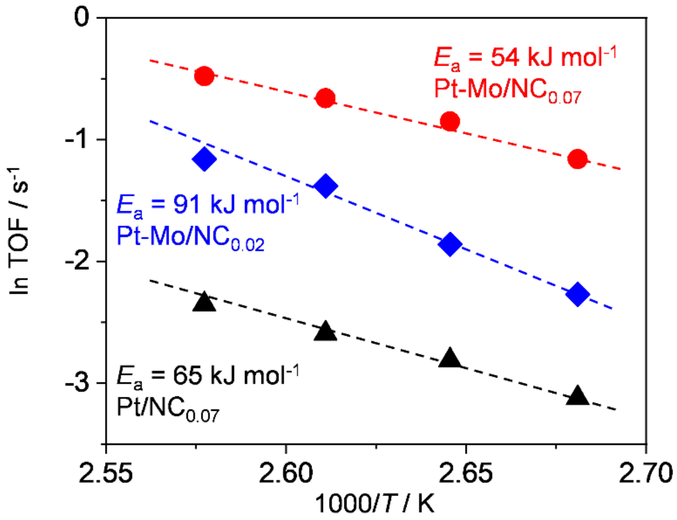

Supplementary Fig. 20. The Arrhenius plots of key Pt-based catalysts for the gas phase formic acid hydrogenation. The HCOOH conversions were restricted to <25% by varying the catalyst weight, to minimize the concentration gradient. TOF: turnover frequency based on the surface Pt, E_a: apparent activation energy.

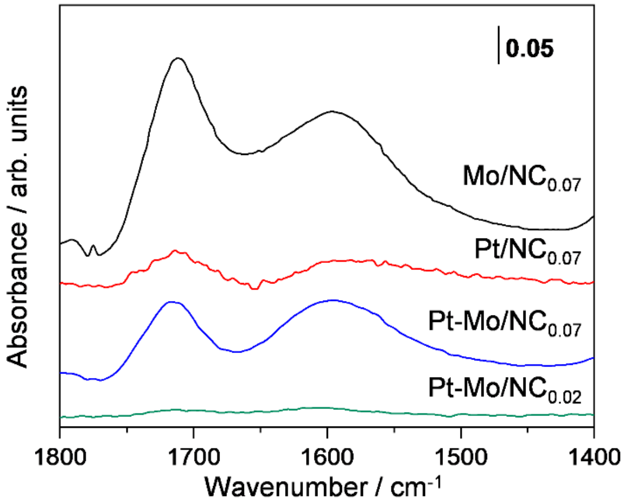

Supplementary Fig. 21. Infrared spectra with HCOOH adsorbed on the mono- and bi-metallic catalysts at the room temperature.

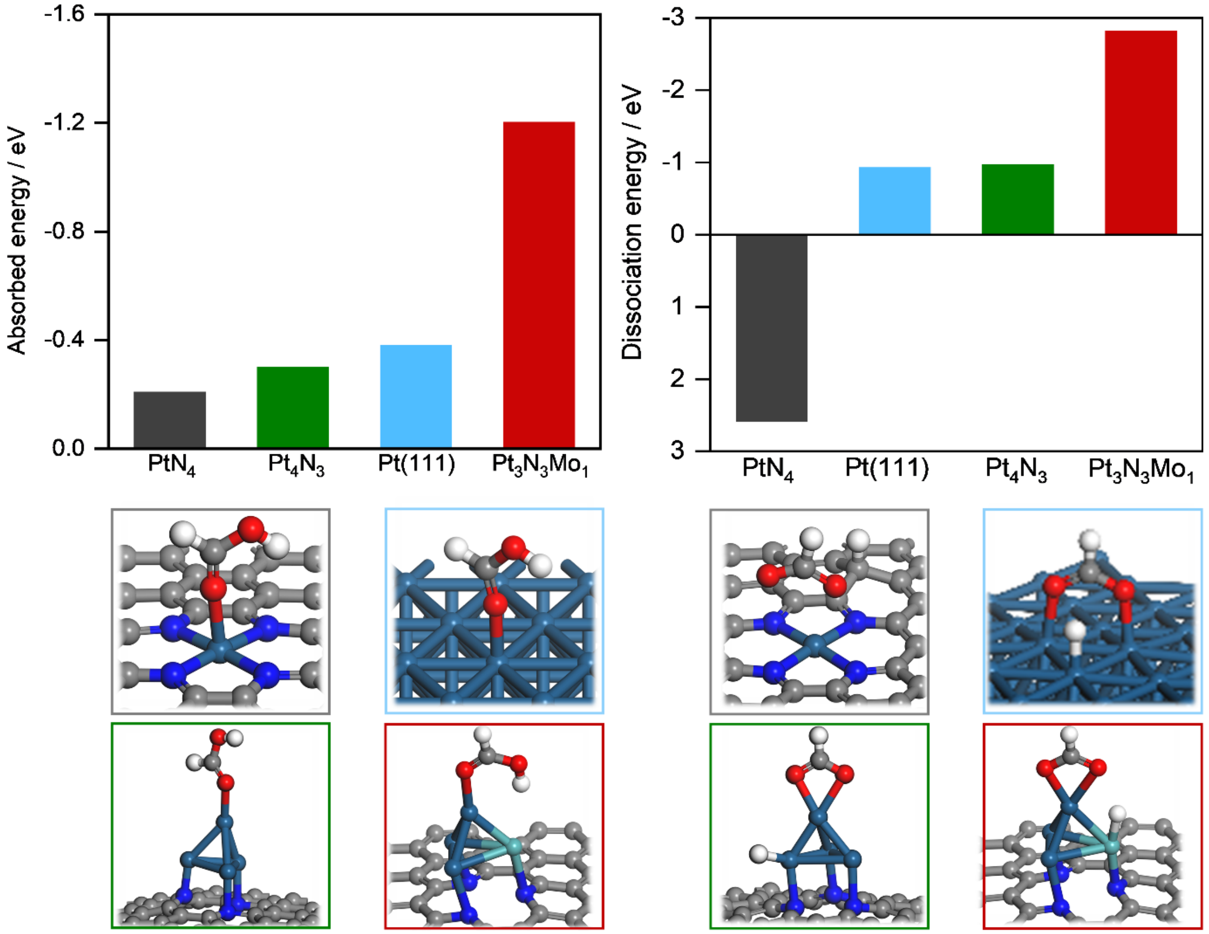

Supplementary Fig. 22. The adsorption and dissociation energies of HCOOH at different Pt model systems, accompanied with the corresponding side-view configurations.

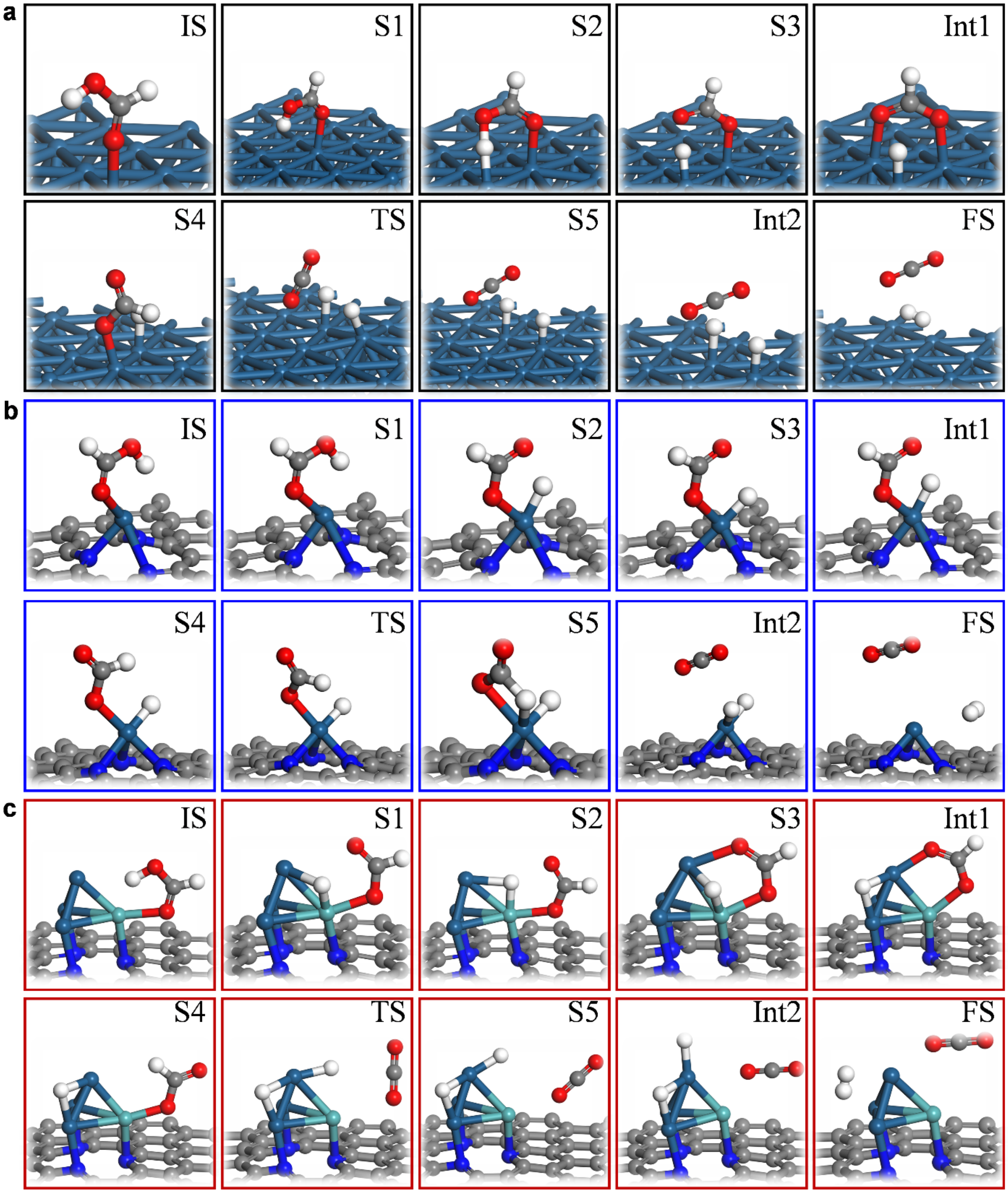

Supplementary Fig. 23 Side view of DFT-optimized adsorption configurations of the intermediates on different catalysts. a, Pt(111), **b**, Pt_1N_3, and **c**, Pt_3MoN_3. All these configurations correspnd to the reaction profiles in Fig. 5.

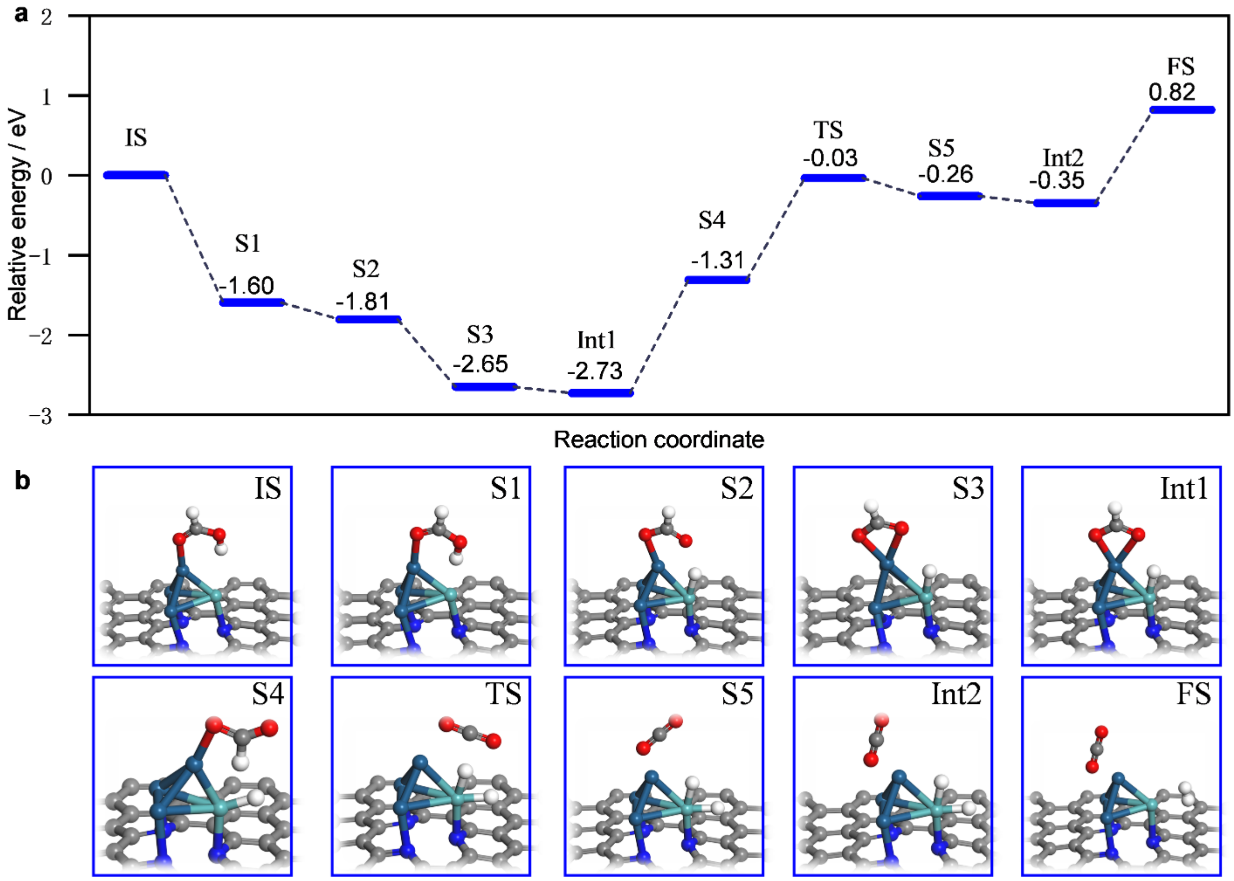

Supplementary Fig. 24. **The reaction coordinate of HCOOH decomposition on $Pt_3Mo_1N_3$ starting from HCOOH adsorption at the top Pt sites. a**, The relative energy, and **b**, the side view of DFT-optimized adsorption configurations of the intermediates.

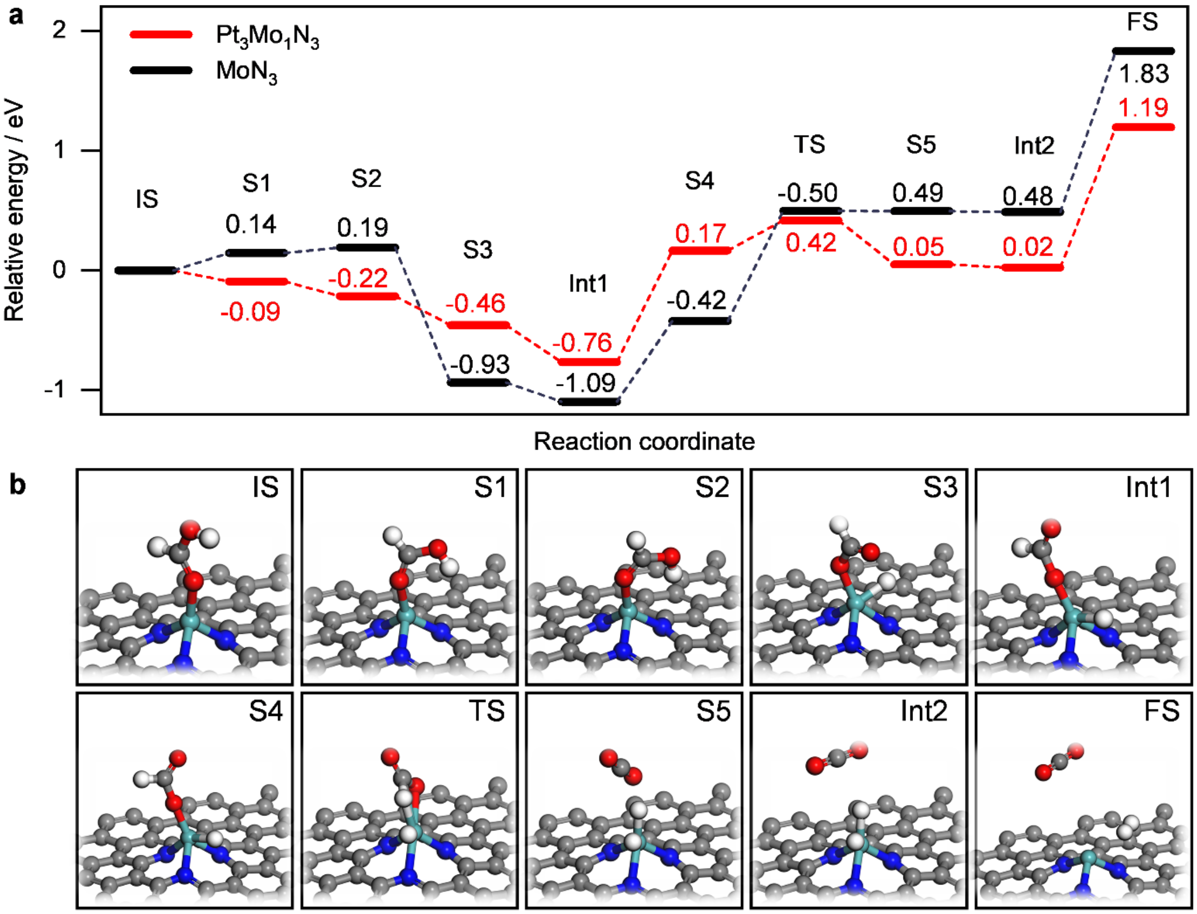

Supplementary Fig. 25. The reaction coordinate of HCOOH decomposition on MoN_3 single-atom model. a, The relative energy, and **b**, the side view of DFT-optimized adsorption configurations of the intermediates. The reaction simulation on $Pt_3Mo_1N_3$ was provided in **a** for reference.

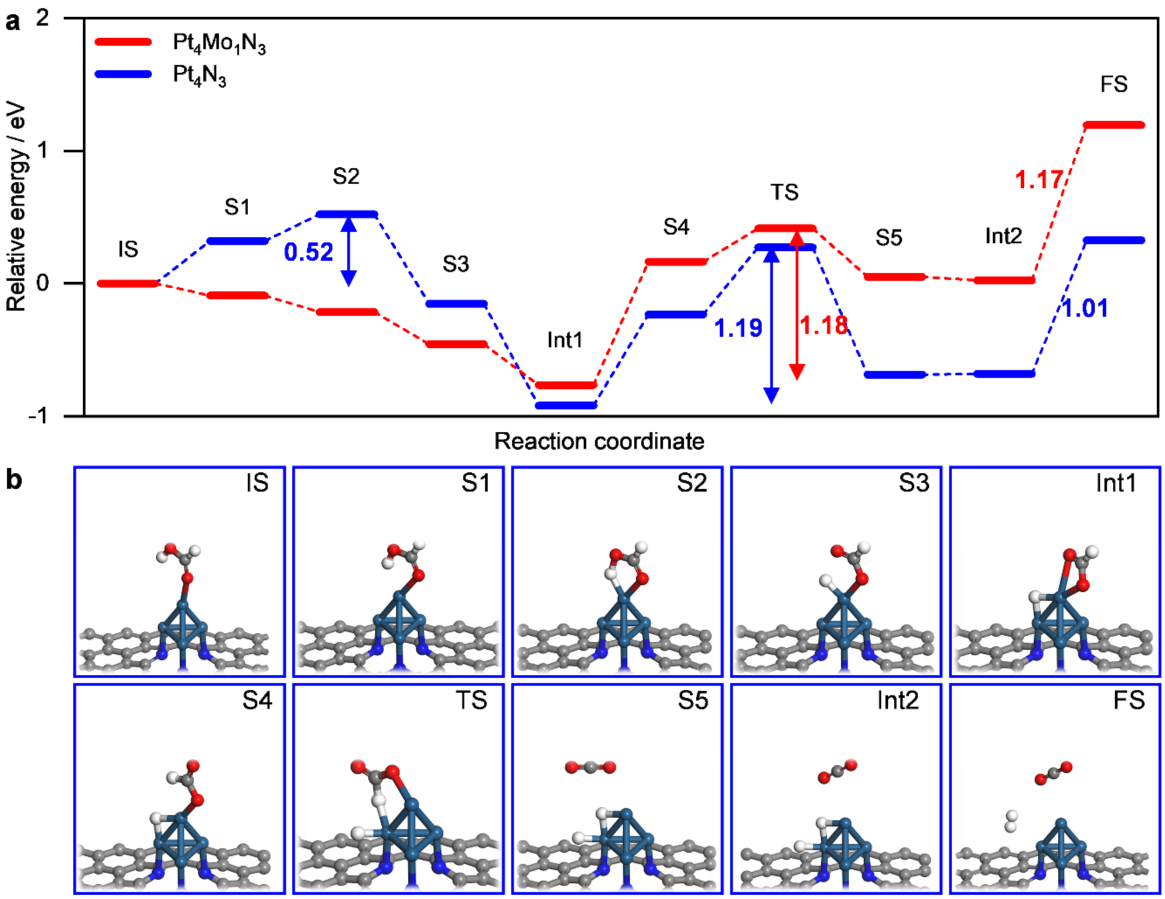

Supplementary Fig. 26. The reaction coordinate of HCOOH decomposition on Pt_4N_3. a, The relative energy, and **b**, the side view of DFT-optimized adsorption configurations of the intermediates. The energy barriers in **a** were highlighted by the bold numbers. The reaction simulation on $Pt_3Mo_1N_3$ was provided in **a** for reference.

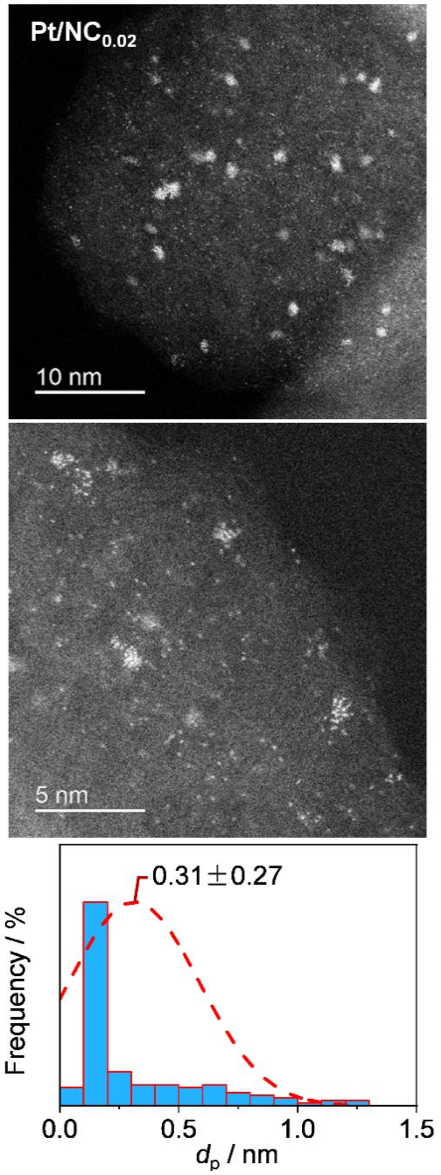

Supplementary Fig. 27. **HAADF-STEM images of Pt/NC$_{0.02}$ and the particle size distributions.** This reference catalyst was prepared following the same recipe as Pt/NC$_{0.07}$ but with a lower reduction temperature of 573 K in order to reach similar particle size distributions as those of Pt-Mo/NC$_{0.07}$.

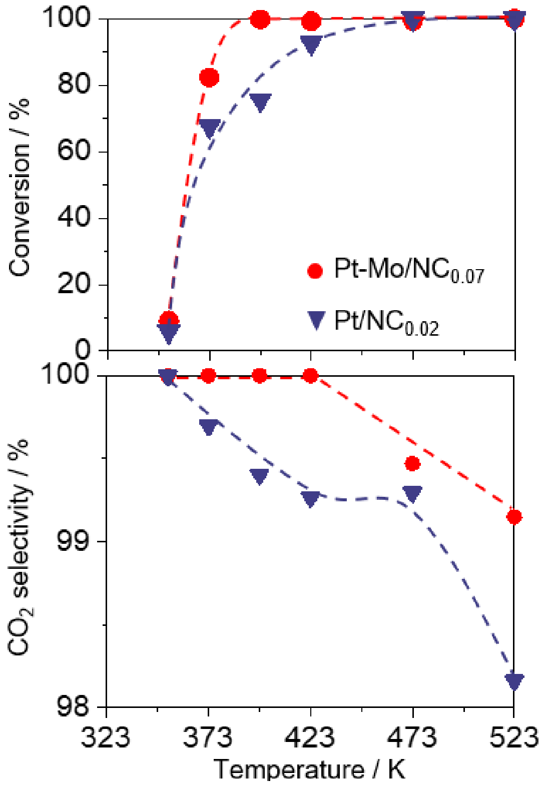

Supplementary Fig. 28. Comparison on the catalytic performance between Pt/NC$_{0.02}$ and Pt-Mo/NC$_{0.07}$ in HCOOH decomposition.

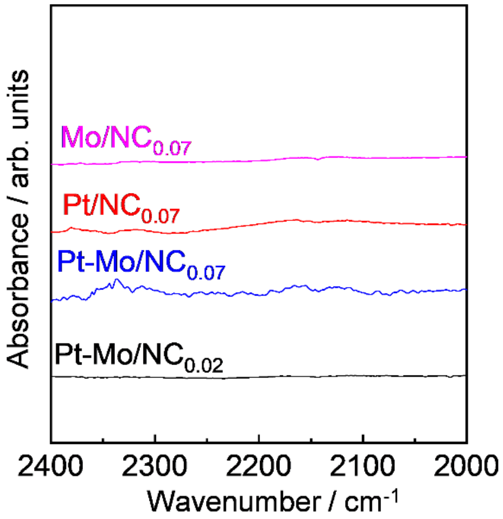

Supplementary Fig. 29. Infrared spectra with CO on the selected catalysts at 77 K. CO chemisorption is difficult on the selected catalysts.

References

1. Bulushev, D.A., Jia, L., Beloshapkin, S., Ross, J.R. Improved hydrogen production from formic acid on a Pd/C catalyst doped by potassium. *Chem. Commun*. **48**, 4184-4186 (2012).

2. Zacharska, M. *et al.* Factors influencing the performance of Pd/C catalysts in the green production of hydrogen from formic acid. *ChemSusChem* **10**, 720-730 (2017).

3. Golub, F.S. *et al.* Boosting hydrogen production from formic acid over Pd catalysts by deposition of N-containing precursors on the carbon support. *Energies* **12**, 3885 (2019).

4. Jia, L. *et al.* Pt nanoclusters stabilized by N-doped carbon nanofibers for hydrogen production from formic acid. *J. Catal*. **307**, 94-102 (2013).

5. Bulushev, D.A. *et al.* Single atoms of Pt-group metals stabilized by N-doped carbon nanofibers for efficient hydrogen production from formic acid. *ACS Catal*. **6**, 3442-3451 (2016).

6. Solymosi, F., Koós, Á., Liliom, N., Ugrai, I. Production of CO-free H_2 from formic acid. A comparative study of the catalytic behavior of Pt metals on a carbon support. *J. Catal.* **279**, 213-219 (2011).

7. Chesnokov, V.V., Kriventsov, V.V., Prosvirin, I.P., Gerasimov, E.Y. Effect of platinum precursor on the properties of Pt/N-graphene catalysts in formic acid decomposition. *Catalysts*, **12**, 1022 (2022).

8. Zacharska, M. *et al.* Support effect for nanosized Au catalysts in hydrogen production from formic acid decomposition. *Catal. Sci. Technol.* **6**, 6853-6860 (2016).

9. Carrales-Alvarado, D.H. *et al.* Selective hydrogen production from formic acid decomposition over Mo carbides supported on carbon materials. *Catal. Sci. Technol.* **10**, 6790-6799 (2020).

10. Zhu, S., Pan, Z., Tao, Y., Chen Y. Low temperature H_2 production from formic acid aqueous solution catalyzed on metal doped Mo_2C. *J. Renew. Mater*. **8**, 939-946 (2020).

11. Wang, N. *et al.* In situ confinement of ultrasmall Pd clusters within nanosized silicalite-1 zeolite for highly efficient catalysis of hydrogen generation. *J. Am. Chem. Soc*. **138**, 7484-7487 (2016).

12. Zhu, Q.-L. *et al.* A solvent-switched in situ confinement approach for immobilizing highly-active ultrafine palladium nanoparticles: boosting catalytic hydrogen evolution. *J. Mater. Chem. A* **6**, 5544-5549 (2018).

13. Hong, W. *et al.* Immobilization of highly active bimetallic PdAu nanoparticles onto nanocarbons for dehydrogenation of formic acid. *J. Mater. Chem. A* **7**, 18835-18839 (2019).

14. Wang, Q., Tsumori, N., Kitta, M., Xu, Q. Fast dehydrogenation of formic acid over palladium nanoparticles immobilized in nitrogen-doped hierarchically porous carbon. *ACS Catal.* **8**, 12041-12045 (2018).

15. Cheng, W. *et al.* Defect-dominated carbon deposited Pd nanoparticles enhanced catalytic performance of formic acid dehydrogenation. *Appl. Surf. Sci*. **597**, 153590 (2022).

16. Leng, Y. *et al.* Cobalt–polypyrrole/melamine-derived Co–N@NC catalysts for efficient base-free formic acid dehydrogenation and formylation of quinolines through transfer hydrogenation. *ACS Appl. Mater. Interface*. **12**, 474-483 (2020).

17. Peng, W.-F. *et al.* Enhanced activity of WO_x-promoted PdNi nanoclusters confined by amino-modified KIT-6 for dehydrogenation of additive-free formic acid. *ACS Sustain. Chem. Eng*. **11**, 1898-1908 (2023).

18. Gao, N. *et al.* Hydrogen-bonded network in interfacial water confer the catalysts with high formic acid decomposition performance. *Appl. Catal. B* **336**, 122913 (2023).

19. Zhang, A., Xia, J., Yao, Q., Lu, Z.-H. Pd-WO_x heterostructures immobilized by MOFs-derived carbon cage for formic acid dehydrogenation. *Appl. Catal. B* **309**, 121278 (2022).

20. Yu, Z. *et al.* Selective dehydrogenation of aqueous formic acid over multifunctional γ-Mo_2N catalysts at a temperature lower than 100 ^{o}C? *Appl. Catal. B* **313**, 121445 (2022).
